# Genotype influences antidepressant discontinuation in a pre-emptive pharmacogenetic testing population

**DOI:** 10.1038/s41397-026-00416-2

**Published:** 2026-05-21

**Authors:** Jordan F. Baye, Natasha J. Petry, Lindsay Hines, Max Weaver, Michael Reinke, Halle Brady, Amanda Massmann, April Schultz

**Affiliations:** 1 Department of Pharmacy Practice, South Dakota State University, College of Pharmacy & Allied Health Professions, Brookings, SD USA; 2 Sanford Imagenetics, Sioux Falls, SD USA; 3 Department of Internal Medicine, University of South Dakota Sanford School of Medicine, Vermillion, SD USA; 4 Department of Pharmacy Practice, North Dakota State University, Fargo, ND USA; 5 Department of Psychiatry and Behavorial Science, University of North Dakota, Grand Forks, ND USA

**Keywords:** Health services, Pharmacogenomics

## Abstract

We performed a retrospective cohort study to evaluate risk of antidepressant discontinuation as a function of CYP2C19 and CYP2D6 phenotype. Patients who participated in an elective genomic screening and had a diagnosis of depression or anxiety with a corresponding antidepressant were analyzed. This resulted in 5 808 patients comprising 8 571 antidepressant orders. Of the total antidepressant orders, 51% of the antidepressants ordered before pharmacogenetic testing were discontinued. A multivariate analysis – accounting for age, medication tenure, allergies, PHQ-9, malaise/fatigue, and medication indication – revealed a significant association between metabolizer status and discontinuation for CYP2C19 increased metabolizers (ultrarapid plus rapid metabolizers) compared to normal metabolizers (n = 4 635) (HR = 1.17 [1.08, 1.27], p < 0.001) for CPIC guideline-recommended medications (i.e., citalopram, escitalopram, sertraline). Stratifying risk by individual drug maintained significant associations for escitalopram (HR = 1.27 [1.1, 1.46], p < 0.05) and sertraline (HR = 1.15 [1.01, 1.31], p < 0.05). While the CYP2D6 decreased metabolizer group (intermediate plus poor metabolizers) did not reach statistical significance for guideline recommended medications (i.e., paroxetine, venlafaxine, vortioxetine), the individual effect for venlafaxine showed an increased risk of discontinuation (HR = 1.23 [1.01, 1.5], p < 0.05). These results suggest phenotypic variations may have variable impact on risk of discontinuation amongst different antidepressant medications, but for escitalopram, sertraline, and venlafaxine, the risk of discontinuation in particular phenotypes should be considered at the time of therapy initiation.

## Introduction

An estimated 21 million adults in the United States -- about 8.3% of the adult population -- live with depression with the majority receiving pharmacotherapy treatment [1]. Selective serotonin reuptake inhibitors (SSRIs) and serotonin/norepinephrine reuptake inhibitors (SNRIs) have been first-line pharmacotherapy options for more than twenty years; however, these medications typically require a minimum of 4-6 weeks for an adequate trial of the treatment. Even with ideal use, over 50% of patients will experience at least one treatment failure and only about 30% will achieve remission on their first agent [2]. Additionally, since multiple medication trials are attempted before achieving an acceptable outcome, many individuals may experience extended periods of unmanaged psychiatric illness.

Premature discontinuation and non-adherence of antidepressant medications can result in unfavourable outcomes in patients being treated for depressive disorders at all stages of treatment. Poor adherence has been associated with decreased treatment response and remission rates, worsening severity, and increased healthcare cost and utilization [3, 4]. Furthermore, around half of all patients will discontinue an antidepressant prematurely with a common reason being side effect burden [5]. Abrupt discontinuation can cause acutely distressing symptoms of antidepressant discontinuation syndrome further perpetuating beliefs that antidepressants cause adverse effects [6]. Following remission of depressive symptoms, some recent guidelines recommend continuation of medication therapy for at least 6 months, and even longer in individuals at higher risk of future episodes [7, 8]. During continued therapy, premature discontinuation has been associated with increased risk for recurrence of future depressive episodes [9].

Current guidelines by the Clinical Pharmacogenetics Implementation Consortium (CPIC) offer pharmacogenetic (PGx) dosing recommendations for several SSRIs/SNRIs [10]. Recommendations focus predominantly on three Cytochrome P450 metabolism genes - *CYP2B6*, *CYP2C19*, and *CYP2D6*. The enzymes produced by these genes metabolize seven different medications: *CYP2C19* – citalopram and escitalopram; *CYP2D6* – fluvoxamine, paroxetine, venlafaxine, and vortioxetine; and *CYP2B6/CYP2C19* – sertraline. These medications experience differential metabolism on the basis of genotype, and per the guidelines, there is a direct genotype-phenotype-outcome association that is both consistent and predictable. Increased enzyme function is associated with lower serum drug concentrations and a corresponding decrease in efficacy. Inversely, decreased CYP450 function is associated with higher serum drug concentrations and a proportional risk of adverse effects. Notably, fluoxetine is also metabolized by CYP2D6, though clinical studies have not discovered differences in clinical outcomes based on phenotype.

With many patients experiencing suboptimal response and remission with a first or second agent, PGx has demonstrated clinical impact amongst individuals with depressive symptoms and major depressive disorder (MDD). Previous studies have shown that with guidance from PGx testing, patients were more likely to switch to a congruent therapy based on their results that led to significant improvement in time to remission or response compared to those that received standard of care [11, 12]. While improvement in symptoms, response, remission, and adverse toxicities has been evaluated in other studies; the impact of PGx testing on antidepressant discontinuation has not been fully explored. Herein, we evaluated real-world data on the effect of genetic variations on therapy discontinuation in patients with depression and anxiety.

## Methods

### Site description and implementation

This study was conducted in a rural, not-for-profit health system located in the upper Great Plains of the U.S. inclusive of South Dakota, North Dakota, Iowa, and Minnesota. The patient biogeographical group is predominantly European, with over 90% identifying as European American. PGx testing was implemented system-wide within our health system in 2014, and *CYP2C19* and *CYP2D6* have been a standard component of PGx testing since that time [13]. The PGx panel has undergone several iterations since inception, though tested alleles have remained current with guideline recommendations from CPIC and tier 1 recommendations from the Association for Molecular Pathology (AMP) [14, 15]. CYP2C19 and CYP2D6 phenotypes are based off the most recent CPIC antidepressant guidelines [10]. Relevant *CYP2C19* alleles common among all iterations of testing were *2, *3, *4, *5, *6, *7, *8, *17. Relevant *CYP2D6* alleles common among all iterations of testing were *2, *3, *4, *5 (gene deletion), *6, *9, *10, *41, with *17 and *29 added shortly after implementation. *CYP2D6* diplotype also accounted for copy number variants, however, did not identify which allele was duplicated.

### Study design

This was a retrospective, single-institution, cohort study. Data was aggregated from a single electronic medical record (EMR) spanning 2011 to 2024. All patients that received PGx testing for a clinical indication were initially evaluated. Of those, patients were included if they had an ICD10 diagnostic code for depression or anxiety and had an order for an antidepressant. The study specifically investigated seven antidepressants - citalopram, escitalopram, fluvoxamine, paroxetine, sertraline, venlafaxine, and vortioxetine. These medications were chosen due to inclusion in CPIC guidelines. Additionally, concomitant use of strong CYP2D6 inhibitors (bupropion, paroxetine, fluoxetine, quinidine, and terbinafine) within 90 days of antidepressant use was incorporated within the analyses to represent clinical phenotype as strong inhibitors have been correlated with enzymatic activity of poor metabolizers [16, 17].

The primary endpoint was an association between antidepressant discontinuation and CYP2D6 and/or CYP2C19 metabolizer status. Specifically, it was hypothesized that increased or decreased CYP2C19 metabolism would have higher discontinuation rates in individuals prescribed citalopram, escitalopram, or sertraline. Similarly, increased or decreased CYP2D6 metabolism was hypothesized to lead to higher discontinuation rates for individuals prescribed fluvoxamine, paroxetine, venlafaxine, or vortioxetine.

### Ethics approval and consent to participate

This study was reviewed and approved by the Sanford Research Institutional Review Board (protocol # 00003589). The requirement for informed consent was waived by the IRB as the study approach utilized only existing data. All methods were performed in accordance with relevant guidelines and regulations.

### Data definitions and censoring

Since discontinuation is not a discrete variable in our EMR, the research team defined “discontinuation” as a break in contiguous prescriptions for each individual medication. Patients could potentially have multiple entries within the data for each antidepressant they had taken before PGx testing. When a given patient had only started taking an antidepressant within 3 days of PGx testing, they were removed as it was deemed not enough time to observe a potential discontinuation. To avoid any possible clinician intervention based on PGx results alone, data was censored at the time of PGx testing. Potential contextual baseline characteristics were evaluated and indexed at the date patients discontinued an antidepressant or the date of PGx testing. Such characteristics included demographics (age, race, sex), specific antidepressant, noted allergies to specific antidepressants, PHQ-9 results, specific antidepressant tenure, and the indication of antidepressant (anxiety only, depression only, anxiety and depression). Diagnoses relevant to discontinuation (history of suicide attempts and/or self-harm, active alcohol or substance abuse, malaise/fatigue, and nausea/vomiting) were also captured if available. These particular diagnoses were chosen based on plausible confounding in discontinuation rates. Missing data was imputed via a K-nearest neighbor model with active diagnoses, sex, and age. PHQ-9 results were missing for 20% of the sample; the imputation model explained 23% of the variance in PHQ-9 whilst maintaining a similar distribution across imputed vs not imputed values.

To account for the impact of drug-drug interactions on genotype-phenotype associations (i.e., phenoconversion), the analysis adjusted the phenotype in the presence of strong drug inhibitors. If the patient was taking bupropion, fluoxetine, paroxetine, terbinafine, and quinidine within 90 days of their antidepressant, they were assigned CYP2D6 poor metabolizer phenotype. Similarly, if the patient was taking fluconazole, fluvoxamine, ticlopidine within 90 days of their antidepressant, they were assigned CYP2C19 poor metabolizer phenotype. While paroxetine undergoes auto-inhibition, we chose to classify and analyze the primary effect according to genotype without reference to its auto-inhibition. However, if paroxetine was acted upon by another inhibiting substate (e.g., bupropion) or was itself impacting another CYP2D6 substrate (e.g., vortioxetine), the analysis considered phenoconversion to be present.

To improve statistical power and simplify the analysis, phenotypes were recategorized from consensus standard definitions (i.e., poor, intermediate, normal, rapid, ultrarapid) to three phenotype groups. CYP2C19 rapid and ultrarapid phenotypes were collapsed into an ‘increased metabolizer’ category. Intermediate and poor phenotypes for both CYP2C19 and CYP2D6 were likewise collapsed into ‘reduced metabolizer’ categories. Since CYP2D6 standardized nomenclature does not define a rapid metabolizer phenotype, CYP2D6 increased metabolizer group included only CYP2D6 ultrarapid phenotypes. The term ‘normal metabolizer’ was retained for the normal metabolizer reference group. Individuals with a non-discrete CYP2D6 phenotype (e.g., a possible range of phenotypes) or indeterminate results were excluded from analysis.

### Data analysis

Fisher’s exact tests were used to preliminarily evaluate the independent effect of decreased versus normal metabolizers and increased versus normal metabolizers for both *CYP2C19* and *CYP2D6*. Baseline patient characteristics were evaluated (Table 1) for initial evaluation of their statistical relevance (p < 0.05) above and beyond their theoretical justification. Statistical significance was calculated using Fisher’s exact test and chi-squared tests for nominal variables and Wilcoxon signed-rank test for linear variables. Controlling for solidified covariates, a series of Cox regression models were fit to isolate the independent effect (hazard ratio) metabolizer status on time to discontinuation whereby normal metabolizers (NM) were treated as a reference group. After initial hypothesis testing of the effect of CYP2D6 and CYP2C19 metabolizer status on discontinuation, additional models were fit for each respective PGx relevant drug to evaluate how it impacts discontinuation in a quasi-sensitivity analysis paradigm (Supplementary Table 1). Given that patients could appear in the data more than once, unique patient identifier (ID) was treated as a random effect. Data were evaluated using R version 4.3.2.

**Table 1:** Baseline characteristics of study population.

Characteristic	Overall, N = 8571	Continue, N = 4201	Discontinue, N = 4370	p-value^*1*^
**CYP2C19, n (%)**				0.036*
*Poor Metabolizer*	680 (7.9)	321 (7.6)	359 (8.2)	
*Intermediate Metabolizer*	1981 (23)	988 (24)	993 (23)	
*Normal Metabolizer*	3213 (37)	1629 (39)	1584 (36)	
*Rapid Metabolizer*	2276 (27)	1065 (25)	1211 (28)	
*Ultrarapid Metabolizer*	421 (4.9)	198 (4.7)	223 (5.1)	
**CYP2D6, n (%)**				0.012*
*Poor Metabolizer*	1036 (12)	463 (11)	573 (13)	
*Intermediate Metabolizer*	3052 (36)	1544 (37)	1508 (35)	
*Normal Metabolizer*	4328 (50)	2116 (50)	2212 (51)	
*Ultrarapid Metabolizer*	155 (1.8)	78 (1.9)	77 (1.8)	
**Age, Median (IQR)**	43 (33 – 57)	46 (35 – 60)	39 (30 – 53)	<0.001***
**Sex, n (%)**				0.85
*Female*	6781 (79)	3320 (79)	3461 (79)	
*Male*	1790 (21)	881 (21)	909 (21)	
**Race, n (%)**				0.48
*African American/Black*	43 (0.5)	18 (0.4)	25 (0.6)	
*American Indian or Alaskan Native*	81 (0.9)	33 (0.8)	48 (1.1)	
*Asian*	30 (0.4)	12 (0.3)	18 (0.4)	
*Caucasian/White*	8376 (98)	4120 (98)	4256 (97)	
*Declined*	17 (0.2)	8 (0.2)	9 (0.2)	
*Hispanic/Latino/White*	3 ( < 0.1)	0 (0)	3 ( < 0.1)	
*Native Hawaiian*	3 ( < 0.1)	2 ( < 0.1)	1 ( < 0.1)	
*Pacific Islander*	3 ( < 0.1)	1 ( < 0.1)	2 ( < 0.1)	
*Unavailable/Unknown*	15 (0.2)	7 (0.2)	8 (0.2)	
**Medication type, n (%)**^**ƚ**^				<0.001***
*Citalopram*	1501 (18)	581 (14)	920 (21)	
*Escitalopram*	2250 (26)	1189 (28)	1061 (24)	
*Paroxetine*	529 (6.2)	227 (5.4)	302 (6.9)	
*Sertraline*	2649 (31)	1410 (34)	1239 (28)	
*Venlafaxine*	1507 (18)	748 (18)	759 (17)	
*Vortioxetine*	135 (1.6)	46 (1.1)	89 (2.0)	
**Medication tenure (years), Median (IQR)**	1.96 (0.56 – 5.07)	3.83 (1.30 – 6.66)	1.02 (0.31 – 2.75)	<0.001***
**Allergy to antidepressant, n (%)**	280 (3.3)	87 (2.1)	193 (4.4)	<0.001***
**PHQ9, Median (IQR)**	6.0 (3.0 – 10.0)	5.0 (2.0 – 9.0)	7.0 (3.0 – 11.0)	<0.001***
**History of suicide attempt and/or self-harm, n (%)**	138 (1.6)	49 (1.2)	89 (2.0)	0.001**
**Active substance abuse disorder, n (%)**	790 (9.2)	364 (8.7)	426 (9.7)	0.083
**Personality disorder, n (%)**	128 (1.5)	54 (1.3)	74 (1.7)	0.12
**Malaise or fatigue, n (%)**	2724 (32)	1227 (29)	1497 (34)	<0.001***
**Active alcohol related disorder, n (%)**	259 (3.0)	129 (3.1)	130 (3.0)	0.80
**Nausea & vomiting, n (%)**	1238 (14)	565 (13)	673 (15)	0.010*
**Indication, n (%)**				<0.001***
*Depression & Anxiety*	3294 (38)	1679 (40)	1615 (37)	
*Anxiety*	4275 (50)	1953 (46)	2322 (53)	
*Depression*	1002 (12)	569 (14)	433 (9.9)	

N is the number of prescriptions.

^1^*p < 0.05; **p < 0.01; ***p < 0.001.

^ƚ^due to lack of prescriptions fluvoxamine is not represented in analyses.

## Results

There were 5 808 patients with 8 571 corresponding antidepressant prescriptions who met inclusion/exclusion criteria for analysis. Baseline characteristics of this population, stratified by discontinuation, are described in Table 1. Of note, fluvoxamine orders were sparse, therefore, it was omitted from analysis. Among all individual antidepressant prescriptions: 4 370 (51%) were discontinued whilst 4 201 (49%) were still actively taking the specific drug by the time of PGx testing. Patients that discontinued their antidepressant tended to be younger (M = 39 vs M = 46, p < 0.001), have a lower specific medication tenure (M = 1.02 years vs M = 3.83, p < 0.001), have a specific antidepressant allergy (4.4% vs 2.1%, p < 0.001), have a higher PHQ-9 score (M = 7 vs M = 5, p < 0.001). Patients that discontinued also had higher rates of comorbidities theorized to influence discontinuation, including: a history of suicide attempts and/or self-harm (2% vs 1.2%, p < 0.001), an active substance abuse disorder (9.7% vs 8.7%, p = 0.083), a personality disorder (1.7% vs 1.3%, p = 0.12), malaise/fatigue (34% vs 29%, p < 0.001), and nausea & vomiting (15% vs 13%, p < 0.01). The indication of antidepressant also differed between those that discontinued and those that did not (p < 0.001). Specifically, those that discontinued had a higher prevalence of anxiety-only (53% vs 46%) and those that continued had higher rates of depression-only (14% vs 9.9%). Furthermore, those that did not discontinue had a higher prevalence of depression *and* anxiety. The specific antidepressant also had different discontinuation rates (p < 0.001).

**Fig. 1:**
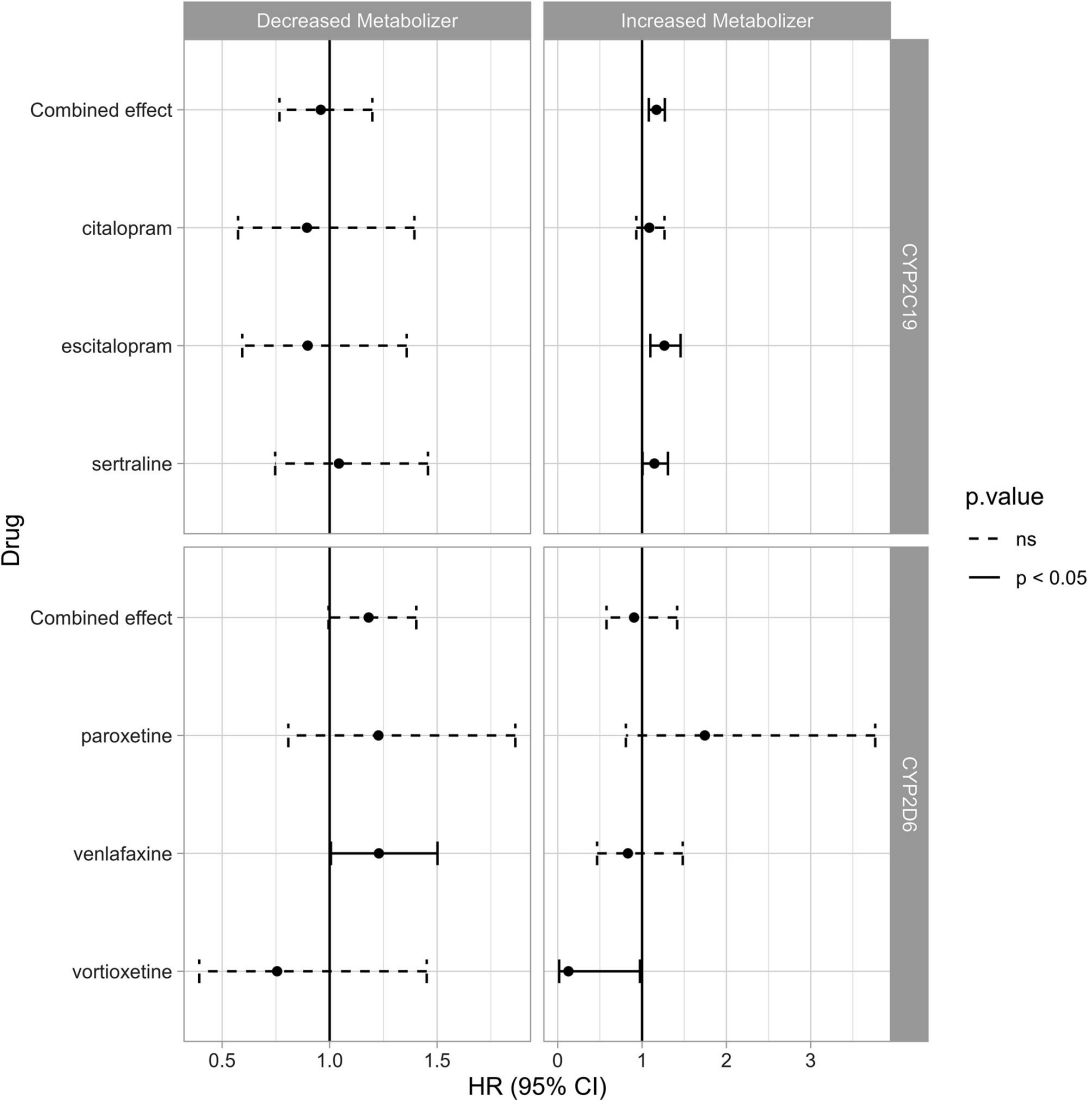
Hazard ratios of metabolizer status and drug type on discontinuation.

To address discontinuation events, a series of Cox regression models were fit for each antidepressant, comparing decreased metabolizers against NMs and comparing increased metabolizers against NMs, for both CYP2D6 and CYP2C19 metabolizer effects. Age, presence of an antidepressant allergy, PHQ-9 scores, a history of malaise/fatigue, and antidepressant indication were to be used as covariates. The isolated hazard ratios of metabolizer status are reported in Table 2 and visualized in Fig. 1. Compared to CYP2C19 NMs, decreased metabolizers did not have differing rates of discontinuation (p > 0.05). CYP2C19 increased metabolizers had increased hazard risk compared to NMs (HR = 1.17 [1.08, 1.27], p < 0.001). When stratifying this risk by each relevant PGx drug, CYP2C19 increased metabolizers had increased risk for patients taking escitalopram (HR = 1.27 [1.1, 1.46], p < 0.05) and sertraline (HR = 1.15, [1.01, 1.31], p < 0.05) compared to NMs, but not for those taking citalopram (HR = 1.09 [0.93, 1.27], p = 0.29). CYP2D6 decreased metabolizers trended toward increased hazard risk of discontinuation compared to NMs but did not reach statistical significance (HR = 1.18 [0.99, 1.4], p = 0.057). Because of the exploratory nature of this study, we chose to evaluate individual medications within this group despite the non-significant primary finding. Evaluation into specific drug effects revealed that venlafaxine increased risk (HR = 1.23 [1.01, 1.5], p < 0.05) while paroxetine and vortioxetine did not (p > 0.05). CYP2D6 increased metabolizers did not have increased risk of discontinuation compared to NMs (HR = 0.91 [0.58, 1.42], p = 0.663). Within this group, patients taking vortioxetine had a lower risk of discontinuation (HR = 0.13 [0.02, 0.98], p < 0.05).

**Table 2:** Cox regression model results.

*PGx Metabolizer Status*	*Drug*	n	Events	*HR (95% CI)*	*p-value*
CYP2C19					
Decreased Metabolizer		2695	1289	0.96 (0.77, 1.2)	0.711
	*citalopram*	629	371	0.89 (0.57, 1.39)	0.623
	*escitalopram*	938	401	0.9 (0.59, 1.36)	0.611
	*sertraline*	1128	517	1.04 (0.75, 1.46)	0.804
Increased Metabolizer		**4632**	**2340**	**1.17 (1.08, 1.27)**	**< 0.001**
	*citalopram*	1088	661	1.09 (0.93, 1.27)	0.291
	*escitalopram*	1626	773	1.27 (1.1, 1.46)	< 0.05
	*sertraline*	1918	906	1.15 (1.01, 1.31)	< 0.05
CYP2D6					
Decreased Metabolizer		1400	759	1.18 (0.99, 1.4)	0.057
	*paroxetine*	335	187	1.23 (0.81, 1.86)	0.337
	*venlafaxine*	982	516	1.23 (1.01, 1.5)	< 0.05
	*vortioxetine*	83	56	0.76 (0.39, 1.45)	0.4
Increased Metabolizer		1146	608	0.91 (0.58, 1.42)	0.663
	*paroxetine*	300	167	1.74 (0.81, 3.77)	0.156
	*venlafaxine*	783	397	0.83 (0.47, 1.48)	0.533
	*vortioxetine*	63	44	0.13 (0.02, 0.98)	< 0.05

Data in this table describes the hazard ratio of metabolizer status on time to discontinuation. The number of discontinuation events (‘events’) among the total medication trials (‘n’). CYP2C19 and CYP2D6 phenotypes were analyzed independently, comparing decreased metabolizer and increased metabolizer groups against the reference group (normal metabolizers). Additional models were fit for each respective PGx relevant drug, which are displayed as the individual drug names under the respective metabolizer groups.

## Discussion

The goal of this study was to determine whether increased or decreased metabolism of CYP2C19 or CYP2D6 was associated with a higher likelihood of discontinuing antidepressant therapy. Results demonstrated that CYP2C19 increased metabolizers – a combination of the standardized rapid metabolizer (RM) and ultrarapid metabolizer (UM) phenotypes – did have a 17% increased risk of discontinuing antidepressant therapy compared to CYP2C19 NMs. Stratifying the data by medication type, escitalopram and sertraline were the key drivers of that effect with a 27% and 15% increased risk of discontinuation, respectively. None of the remaining phenotype groups (CYP2C19 decreased metabolism, CYP2D6 increased metabolism, CYP2D6 decreased metabolism) addressed in this study were statistically associated with discontinuation, though CYP2D6 decreased metabolism trended toward significance. The number of patients taking paroxetine and vortioxetine was notably smaller than the other four medications in our analysis, and this discrepancy may have influenced the power and perhaps affected the combined outcome for CYP2D6 decreased metabolizers. A larger data set may be needed to identify a significant outcome within this group.

A clinically relevant increase in the risk of antidepressant discontinuation was discovered for CYP2C19 increased metabolizers, and while the data shows consistent direction of effect among all three substrates (citalopram, escitalopram, sertraline), only sertraline and escitalopram showed a statistically significant difference. Citalopram had the smallest magnitude of effect (HR = 1.09 [0.93, 1.27], p = 0.26), and it seems reasonable to conclude that the effect would not be considered clinically relevant even if statistical significance was established in a larger data set. For escitalopram and sertraline, these data indicate that higher metabolism increases the risk of medication discontinuation. Increased risk of discontinuation for escitalopram is consistent with a previous study by Jukic and colleagues, which assessed the effect of *CYP2C19* genotype on exposure and therapeutic failure in 4 228 patients treated with escitalopram [17]. In this study, the authors found the frequency of switching from escitalopram to another antidepressant was higher for CYP2C19 RMs and UMs groups compared to NMs. Odds of switching was 1.6 (p = 0.003) and 3.0 (p < 0.001) for RMs and UMs, respectively. Conversely, Wong and colleagues found CYP2C19 “fast metabolizers” (i.e., combined UM + RM group) were less likely to switch from escitalopram to another antidepressant versus NMs (OR = 0.601, 95% CI 0.374-0.966, p = 0.036) [18]. These data were collected from the UK Biobank that included 3 012 individuals taking escitalopram; there are notable similarities in population size/demographics, phenotype distribution, and approach between their study and the present investigation. One potentially important difference is that Wong et al analyzed medication discontinuation and medication switching as different types of outcomes, whereas our study did not distinguish between these. This small but important difference may be responsible for the differences seen between the two studies. Wong et al also evaluated sertraline in their analysis; likewise they found no association for sertraline in CYP2C19 increased metabolizers. Similar outcomes were found for sertraline in two other studies [19, 20]. Thus, our study is to our knowledge the first to identify increased risk of discontinuation for sertraline in CYP2C19 increased metabolizers. Of note, for both sertraline and escitalopram, there is limited agreement amongst several different studies. Differences in population stratification, size, and scope, may limit meaningful comparisons. Importantly, there seems to be no standardized or consensus definition for discontinuation, and some studies chose to stratify discontinuation-like outcomes into separate categories (e.g., medication switch vs dose change vs discontinuation). Future studies evaluating discontinuation event should be mindful of these differences and consider evaluating the individual outcomes in aggregate as well.

Whereas our study did not correlate *CYP2C19* decreased metabolism and discontinuation in all medications, it is noteworthy that several other studies have found this association. Jukic and colleagues found a 3.3-fold higher odds of escitalopram discontinuation in the poor metabolizer (PM) group versus NM group (p < 0.01) [21]. Other studies have replicated these findings. A study by Aldrich and colleagues, which assessed response and tolerability of escitalopram in 180 pediatric patients with depression or anxiety, found a combined group of CYP2C19 intermediate metabolizers (IMs) and poor metabolizers as significantly more likely to discontinue escitalopram/citalopram than NMs (p = 0.007) [22]. Further, Thiele and colleagues performed a retrospective cohort study of 17 297 individuals with depression in Denmark. In a subset of children and adolescents, they found CYP2C19 PMs taking citalopram or escitalopram were 64% more likely to switch medications (RR = 1.64, 95% CI 1.1-2.43) than NMs [20], though this effect was not replicated in their young adult and adult cohorts. It is unclear why the discontinuation effect for CYP2C19 decreased metabolizers was not seen in our cohort. Despite utilizing a large population and controlling for known confounders, combined and individual analyses in our study consistently showed CYP2C19 decreased metabolizers did not have increased risk of discontinuation with citalopram, escitalopram, and/or sertraline (both in aggregate and individual analyses). One possible explanation is that the discontinuation effect of the PM group alone may have been obscured by combining it with IMs. This effect (i.e., PMs were associated with discontinuation whereas IMs were not) was seen in both the Aldrich and Thiele studies noted above. One suggestion for future studies, especially those investigating large datasets, would be to stratify results by consensus phenotypes so that the impact of substantial gain- or loss-of-function can be clearly identified.

Our study also did not identify a discontinuation effect for CYP2D6 decreased metabolizers in the combined medication analysis, though individual analysis of venlafaxine was associated with a 23% increase risk of discontinuation (HR = 1.23 [95% CI, 1.01-1.5]). This is, to our knowledge, the first report that venlafaxine discontinuation may be influenced by CYP2D6 phenotype. Previous studies have investigated CYP2D6 genotype and risk of discontinuation with varying outcomes. Mulder and colleagues found CYP2D6 PMs taking an antidepressant that was at least partially metabolized by CYP2D6 had a higher risk of switching medications (RR 3.5, [95% CI 1.52-8.1]); and while the study did include venlafaxine, all 11 antidepressants in the trial were grouped together for the analysis and discontinuation of individual antidepressants was not assessed [23]. Another study by Brouwer and colleagues analyzed the rate of discontinuation/switching of nearly 3000 participants within the Netherlands Study of Depression and Anxiety (NESDA) longitudinal cohort [19]. Their study did not identify statistically significant associations with either CYP2C19 or CYP2D6 phenotypes versus the corresponding antidepressant medications. Individual medication outcomes were not reported. The present study has a number of advantages over previous studies assessing CYP2D6 impact on discontinuation. First, our study was significantly larger than many studies to date, thereby increasing statistical power. Second, our analysis addressed phenoconversion effects in CYP2D6, which allowed us to account for not just genotypic assignments but the impact of drug-drug interactions when assigning phenotype. Regardless of these advantages, we were unable to find a difference in discontinuation risk with paroxetine despite paroxetine having an effect size similar to venlafaxine in the CYP2D6 decreased metabolizer group. Given that the number of patients taking paroxetine was substantially smaller than venlafaxine, a larger population may have led to a significant finding.

A decrease in vortioxetine discontinuation for CYP2D6 decreased metabolism was also discovered in our analysis. While the number of participants was small relative to the other groups in our study, nonetheless a strong effect size and statistically significant outcome was observed. We are unaware of any previous studies addressing vortioxetine discontinuation, and our findings serve as a starting point for future discovery.

Our study has several limitations. First, because this study is based on a clinical cohort from a single institution, there are inherent biases and limits to the generalizability of the findings. Retrospective clinical data is prone to missing data, and our study was no exception. In order to account for missing data, we utilized a validated imputation method (K-nearest neighbor model) and tested assumptions of the model to confirm validity. Because retrospective studies lack randomization, the model controlled for multiple covariates. We started with a set of a priori conditions expected to influence study outcomes and incorporated additional variables statistically associated with discontinuation. This approach does increase potential for bias, though we attempted to limit bias by establishing the analytic framework prior to analysis and subsequently validating clinical interpretation separately (statistician vs clinician). Second, our institution covers several states across the central United States, and our patient population is ethnically relatively homogenous. Ninety-eight percent of the population in this study identified as “white” or “Caucasian”. While this strengthens internal validity and inherently decreases ethnicity as a variable, these data should be applied cautiously to non-white populations. Third, as we did not examine the nature of discontinuation, it is unclear how often discontinuation was premature or representative of therapeutic failure. Accordingly, the clinical impact of these findings is not entirely certain. Fourth, CPIC guidelines provide guidance for both *CYP2C19* and *CYP2B6*, whereas the present study addresses only *CYP2C19*. CYP2B6 is expected to display a normal distribution with relatively rare UM and PM frequencies ( < 5%) in our patient population. As a result, the CYP2C19 outcomes are not expected to be profoundly impacted. Finally, this study did not consistently identify increased rate of discontinuation in all phenotypes and all studied medications. While *CYP2C19* increased metabolizers did show this association, *CYP2D6* increased, *CYP2D6* decreased, and CYP2C19 decreased metabolizers were no more likely to lead to discontinuation than the reference cohort. The inconsistent effect is challenging to explain, given the myriad of factors that impact clinical decision-making. Larger, prospective, randomized studies are needed to better control for outcomes related to medication discontinuation.

## Conclusions

Findings from the current investigation imply CYP2C19 phenotype is associated with differential risk of discontinuation. Subgroup analysis identified escitalopram and sertraline as the key drivers of this effect. Given similar findings for escitalopram in previous studies, these data emphasize a very likely association between increased metabolism and escitalopram discontinuation. While statistically significant outcomes were not found in other phenotypes for aggregate medication groups, discontinuation effects were noted for individual medications -- venlafaxine in CYP2D6 decreased metabolizers and vortioxetine for CYP2D6 increased metabolizers. These latter outcomes are hypothesis-generating for future studies. The relationship between phenotype and antidepressant discontinuation remains poorly understood, and this study sheds additional light on potential implications for medication management and offers new direction for study. Furthermore, these findings support clinical benefits of PGx testing, suggesting this approach could help avoid medications that are more likely to be discontinued prematurely. Future studies should consider limiting data aggregation (i.e., grouping medications or phenotypes) and standardizing terminology used for discontinuation.

## Supplementary information

Supplementary Table 1

## Data Availability

The datasets generated and analysed during the current study are not publicly available due to institutional policy but are available from the corresponding author on reasonable request.
