# Supplementary material for: Genotype influences antidepressant discontinuation in a pre-emptive pharmacogenetic testing population
Source: Pharmacogenomics J. 2026 May 21;26(3):22. doi: 10.1038/s41397-026-00416-2 (PMC13193940; doi:10.1038/s41397-026-00416-2)
Supplement: Supplementary file 1 — Supplementary Table 1 [file 41397_2026_416_MOESM1_ESM.docx]

| ***Metabolizer status*** | ***Drug*** | ***Age*** | ***Allergy*** | ***PHQ-9*** | ***Malaise/fatigue*** | ***Indication - anxiety*** | ***Indication - depression*** | ***Metabolizer status compared to NM*** |
| --- | --- | --- | --- | --- | --- | --- | --- | --- |
| CYP2C19 | | | | | | | | |
| Decreased Metabolizer | combined effect | 0.89 (0.88, 0.91) ** | 1.42 (1.07, 1.87) * | 1.04 (1.03, 1.05) ** | 1.06 (0.95, 1.20) | 1.50 (1.33, 1.70) ** | 1.37 (1.13, 1.66) * | 0.96 (0.77, 1.20) |
|  | citalopram | 0.87 (0.83, 0.90) ** | 1.08 (0.57, 2.03) | 1.05 (1.03, 1.07) ** | 0.99 (0.79, 1.23) | 1.44 (1.13, 1.83) * | 1.38 (0.99, 1.93) | 0.89 (0.57, 1.39) |
|  | escitalopram | 0.89 (0.86, 0.92) ** | 1.24 (0.76, 2.03) | 1.03 (1.01, 1.05) ** | 1.17 (0.95, 1.44) | 1.41 (1.14, 1.75) * | 1.33 (0.93, 1.91) | 0.90 (0.59, 1.36) |
|  | sertraline | 0.91 (0.88, 0.94) ** | 1.85 (1.22, 2.81) * | 1.04 (1.02, 1.06) ** | 1.06 (0.88, 1.28) | 1.67 (1.37, 2.03) ** | 1.34 (0.97, 1.86) | 1.04 (0.75, 1.46) |
| Increased Metabolizer | combined effect | 0.89 (0.88, 0.91) ** | 1.55 (1.26, 1.89) ** | 1.03 (1.03, 1.04) ** | 1.04 (0.95, 1.13) | 1.43 (1.30, 1.57) ** | 1.32 (1.14, 1.52) ** | 1.17 (1.08, 1.27) ** |
|  | citalopram | 0.86 (0.84, 0.89) ** | 1.39 (0.91, 2.11) | 1.04 (1.03, 1.05) ** | 1.03 (0.87, 1.21) | 1.35 (1.13, 1.61) ** | 1.25 (0.97, 1.62) | 1.09 (0.93, 1.27) |
|  | escitalopram | 0.89 (0.87, 0.92) ** | 1.45 (1.02, 2.04) * | 1.02 (1.01, 1.03) * | 1.14 (0.98, 1.32) | 1.36 (1.16, 1.59) ** | 1.34 (1.04, 1.73) * | 1.27 (1.10, 1.46) * |
|  | sertraline | 0.91 (0.89, 0.93) ** | 1.79 (1.31, 2.45) ** | 1.04 (1.03, 1.05) ** | 0.97 (0.84, 1.12) | 1.55 (1.33, 1.79) ** | 1.31 (1.02, 1.67) * | 1.15 (1.01, 1.31) * |
| CYP2D6 | | | | | | | | |
| Decreased Metabolizer | combined effect | 0.86 (0.84, 0.89) ** | 1.80 (1.26, 2.56) * | 1.02 (1.01, 1.03) * | 1.10 (0.95, 1.28) | 1.31 (1.12, 1.53) ** | 1.00 (0.77, 1.31) | 1.18 (0.99, 1.40) |
|  | paroxetine | 0.83 (0.79, 0.87) ** | 2.81 (1.46, 5.39) * | 1.01 (0.98, 1.04) | 1.05 (0.77, 1.44) | 1.71 (1.23, 2.37) * | 1.96 (1.09, 3.50) * | 1.23 (0.81, 1.86) |
|  | venlafaxine | 0.86 (0.83, 0.89) ** | 1.47 (0.92, 2.34) | 1.02 (1.00, 1.03) * | 1.08 (0.91, 1.29) | 1.27 (1.05, 1.53) * | 0.85 (0.62, 1.18) | 1.23 (1.01, 1.50) * |
|  | vortioxetine | 1.06 (0.94, 1.19) | 2.78 (0.95, 8.19) | 1.01 (0.96, 1.07) | 1.13 (0.63, 2.05) | 1.99 (0.96, 4.13) | 2.35 (0.88, 6.26) | 0.76 (0.39, 1.45) |
| Increased Metabolizer | combined effect | 0.86 (0.83, 0.88) ** | 2.31 (1.55, 3.43) ** | 1.02 (1.01, 1.04) * | 1.10 (0.93, 1.30) | 1.44 (1.21, 1.71) ** | 0.99 (0.73, 1.34) | 0.91 (0.58, 1.42) |
|  | paroxetine | 0.82 (0.78, 0.87) ** | 2.38 (1.15, 4.93) * | 1.01 (0.98, 1.04) | 0.97 (0.69, 1.37) | 1.80 (1.26, 2.56) * | 1.97 (1.05, 3.68) * | 1.74 (0.81, 3.77) |
|  | venlafaxine | 0.86 (0.82, 0.89) ** | 2.34 (1.39, 3.95) * | 1.02 (1.00, 1.04) * | 1.06 (0.87, 1.30) | 1.43 (1.15, 1.77) * | 0.83 (0.58, 1.20) | 0.83 (0.47, 1.48) |
|  | vortioxetine | 1.04 (0.90, 1.20) | 2.74 (0.78, 9.67) | 0.99 (0.93, 1.06) | 1.62 (0.83, 3.17) | 2.27 (0.95, 5.40) | 2.57 (0.88, 7.55) | 0.13 (0.02, 0.98) * |
